# Inducible expression of *F48C1.9* encoding a nematode specific secreted peptide in the adult epidermis upon *Drechmeria* fungal infection

**DOI:** 10.17912/micropub.biology.000090

**Published:** 2019-02-08

**Authors:** Shizue Omi, Nathalie Pujol

**Affiliations:** 1 Centre d'Immunologie de Marseille-Luminy (CIML), Aix-Marseille University, UM2, INSERM U1104, CNRS UMR7280, 13288 Marseille, France.

**Figure 1:**
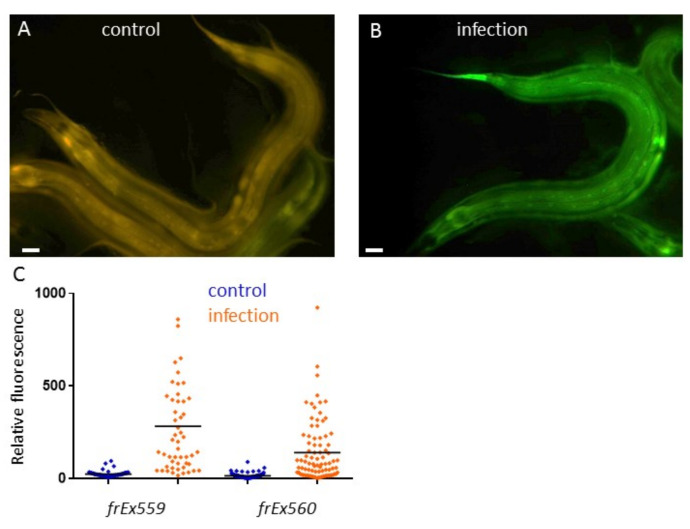
**Inducible expression of *F48C1.9* in the adult epidermis upon fungal infection**. Young adult transgenic worms, carrying both *F48C1.9*p::GFP and *col-12*p::DsRed reporters were infected (infection) or not (control) for 18 hours at 25° C with the fungus *Drechmeria coniospora*, as described in (Pujol et al., 2008a). Representative images with a long pass GFP filter allowing simultaneous visualization of both red and green fluorescence of IG1514 *frEx559* (A-B; bar, 10 µm.) and quantification of relative green fluorescence with the Copas Biosort of this strain (left) and a second independent strain IG1515 *frEx560* (C; each point is a worm, bar indicates mean).

## Description

Inducible immune responses are ubiquitous features of animal defences against infection. *D. coniospora* is a natural pathogen of nematode. It produces spore that attach to specific glycans on the worm’s cuticle surface coat (Rouger et al., 2014), pierce it and send hyphae throughout the organism. This triggers the rapid induction in the epidermis of genes from the *nlp* (for neuro-peptide-like protein) and *cnc* (caenacin) families. These genes encode structurally-related antimicrobial peptides (AMPs) (Pujol et al., 2012); their over-expression can lead to an increased resistance to infection are likely to be important in nature for the survival of worms (Taffoni and Pujol, 2015). Other genes are up-regulated after infection, including *F48C1.9* encoding for a nematode specific small secreted peptide (Pujol et al., 2008b). To monitor its expression, we made transgenic strains containing the transcriptional reporter *F48C1.9*p::GFP with the co-injection marker *col-12*p::DsRed, which is constitutively expressed in the epidermis (Pujol et al., 2008a). Upon infection with *D. coniospora,* an induction of the GFP was observed in the epidermis of the worm (Figure 1). This work suggest that another uncharacterized peptide encoding gene is part of the immune response of the worm epidermis.

## Reagents

Constructs: *F48C1.9*p::GFP was obtained by Gibson fusion of 1.1 kb of the *F48C1.9* promoter in pPD95.75 amplified with primers CACAACGATGGATACGCTAAaatcaaattatgacgtgatgcc and GTTCTTCTCCTTTACTCATgtttgttgaagatctgatctg. Injected at 80 ng/µl together with *col-12*p::DsRed (Pujol et al, 2008a) at 20 ng/µl. Two independent transgenic strains were obtained IG1514 *frEx559* and IG1515 *frEx560[F48C1.9*p::GFP; *col-12*p::DsRed*.].*
